# Adult-Onset PLA2G6-Associated Parkinsonism With Claval Hypertrophy: A Rare Radiological–Genetic Association

**DOI:** 10.7759/cureus.102346

**Published:** 2026-01-26

**Authors:** Biswamohan Mishra, Om Mishra, Manoj Kumar Nayak, Nikhilesh Pradhan, Pradosh Kumar Sarangi

**Affiliations:** 1 Neurology, Kalinga Institute of Medical Sciences, Bhubaneswar, IND; 2 Radiodiagnosis, All India Institute of Medical Sciences, Bhubaneswar, IND; 3 Radiodiagnosis, All India Institute of Medical Sciences, Deoghar, IND

**Keywords:** adult-onset dystonia-parkinsonism, atypical brain mri findings, claval hypertrophy, p.arg741gln mutation, pla2g6-associated neurodegeneration, spastic paraparesis

## Abstract

A man in his 20s presented with rapidly progressive spastic parkinsonism, right-hand focal dystonia, memory impairment, and behavioural changes over eight months, resulting in complete dependence. Examination showed spasticity, rigidity, bradykinesia, ankle clonus, postural instability, slowed saccades, and cognitive deficits (Mini-Mental State Examination 25/30, Montreal Cognitive Assessment 20/30). Brain magnetic resonance imaging revealed mild cerebellar atrophy, claval hypertrophy, and minimal lentiform susceptibility blooming. Whole-exome sequencing identified a heterozygous pathogenic PLA2G6 variant (c.2222G>A; p.Arg741Gln). No family history was present. Symptomatic treatment with levodopa/carbidopa, trihexyphenidyl, and baclofen yielded partial initial benefit but limited sustained response over six months of follow-up. This case expands the adult-onset PLA2G6-associated neurodegeneration phenotype by combining aggressive progression, early cognitive involvement, and rare infantile-like neuroimaging features with minimal iron deposition.

## Introduction

PLA2G6-associated neurodegeneration (PLAN) is a rare autosomal recessive disorder caused by mutations in the *PLA2G6* gene, which encodes a calcium-independent phospholipase A2 essential for phospholipid remodelling and mitochondrial integrity [1,2]. The clinical spectrum ranges from infantile neuroaxonal dystrophy, characterised by rapid progression and prominent brain iron accumulation, to adult-onset dystonia-parkinsonism (PARK14) with slower evolution, variable dystonia, parkinsonism, spasticity, and cognitive decline [3].

Adult-onset cases often lack classic iron deposition and may mimic other hereditary spastic paraplegias or Parkinsonian syndromes [4]. Neuroimaging features such as claval hypertrophy are considered a hallmark sign of the infantile form and are seldom described in adults [5]. We report a case of aggressive adult-onset PLAN combining rapid progression, early cognitive-behavioural involvement, focal dystonia, and unusual neuroimaging findings, illustrating the expanding phenotypic heterogeneity of this disorder and the diagnostic value of early genetic testing.

## Case presentation

A 21-year-old male, born of a non-consanguineous marriage with normal birth and developmental history, presented with an 8-month history of progressive tightness in all four limbs and difficulty walking, eventually requiring assistance. Over the past 3-4 months, he developed memory impairment and occasional aggressive behaviour. In the last month, his gait slowed further, and he became dependent on family members for daily activities. There was no history of seizure, sensory symptoms, or tremor. The patient was the second of four siblings (two sisters and one brother), all of whom are neurologically healthy. No family history of similar neurological illness was reported among siblings, parents, or grandparents, all of whom are alive and unaffected.

On examination, the patient was conscious, cooperative, and of average build. Cognitive scores were Mini-Mental State Examination (MMSE) score [6] of 25/30, a Montreal Cognitive Assessment (MoCA) Version 7.1 score [7] of 20/30, and an Addenbrooke’s Cognitive Examination (ACE-III) score [8] of 68/100, with major deficits in the memory domain. Neurological examination revealed spasticity in all four limbs, brisk reflexes, bilateral ankle clonus, extensor plantar responses, and Medical Research Council (MRC) [9] grade 5/5 power in all limbs except for right ankle dorsiflexion (4-/5) (Videos 1-2). Sensory examination was normal. He had axial and appendicular rigidity, bradykinesia, dystonia of the right hand and postural instability (Videos 2-3). Saccades were slowed on rightward gaze (Video 1). MDS UPDRS (Movement Disorder Society - Unified Parkinson's Disease Rating Scale) score [10] was 112.

**Video 1:** Clinical examination demonstrating extra-ocular movements, muscle tone, power, and reflexes of the patient

**Video 2:** Clinical video demonstrating clonus, tremor, bradykinesia, finger tapping, pronation–supination movements, and toe tapping performed by the patient

**Video 3:** Clinical video demonstrating the gait characteristics of the patient

Primary clinical considerations included complicated hereditary spastic paraparesis (HSP), spinocerebellar ataxia (SCA), Wilson’s disease, adrenoleukodystrophy or metachromatic leukodystrophy and neurodegeneration with brain iron accumulation (NBIA). Routine haematological, biochemical, and metabolic investigations were unremarkable (Table 1).

**Table 1:** Initial investigations performed after hospitalisation in the present case

Parameter (Normal Range)	Value
Haemoglobin (Hb) (13-17 g/dL)	16.1
White Blood Cell (4000-10000/µL)	7319
Platelets count (130-400) × 10^9^/L	2.76
Neutrophils (%)	50.10
Lymphocytes (%)	38
PT (11-16s)	14.60
International Normalized Ratio (INR)	1.09
aPTT (24-35s)	28.10
ESR (mm/hr)	26
CRP Quantitative (<5 mg/L)	4.48
Urea (17-49mg/dL)	26
Creatinine (0.7-1.2mg/dL)	0.9
Uric acid (3.4-7mg/dL)	5.1
Calcium (8.4-10.2mg/dL)	9.1
Phosphorous (2.5-4.5mg/dL)	3.1
Sodium(135-145mmol/L)	145
Potassium (3.5-5.1mmol/L)	4.3
Total Bilirubin (0-1mg/dL)	0.26
Direct Bilirubin (0-0.2mg/dL)	0.11
Indirect Bilirubin (0-0.9mg/dL)	0.15
ALT (<41) /AST (<42)	28.2/23.5
ALP (55-149U/L)	88
Total Protein/ Albumin	6.56/3.85
Serum Ceruloplasmin (22-61mg/dL)	34.0
Serum Copper (80-170ug/mL)	152
Serum Ferritin (30-250 ng/mL)	56.4
Total Cholesterol (<200 mg/dL)	175
Triglycerides (<150 mg/dL)	77
VLDL (0-40 mg/dL)	15.4
LDL (<130 mg/dL)	115.6
HIV/HBsAg/HCV serologies	Negative
Thyroid Stimulating Hormone (TSH) (0.27-4.2 uIU/mL)	2.528
Total T3 (0.82-2.13 ng/mL)	131
Total T4 (5.1-14 ug/mL)	7.8
Vitamin D3 (10-44 ng/mL)	24.8
CK (39-308)	68
Vitamin B12 (197-771 pg/mL)	295
Serum folate (3.1-17.5 ng/mL)	3.86
Serum lactate (<2 mmol/L)	1.2
24-hour urinary copper (3-50 mcg/24 hour)	53.14
Serum Ammonia (10-47 µmol/L)	46
Plasma Lactate (4.5 to 119.8 mg/dL)	18.0
Serum Ferritin (30-200 ng/mL)	38 ng/mL
Fasting Plasma Glucose (FBS) mg/dL	78
Post Prandial Plasma Glucose (PPBS) mg/dL	96
HbA1C	4.6%
Peripheral smear for acanthocytes	Negative
Urine Pus cells	1-2
Urine Protein/Ketone/Sugar/Sediments	Negative
Cerebrospinal fluid (CSF) analysis	Total cell count – 5, all lymphocytes, protein 38 mg/dL, glucose 78 mg/dL, culture - sterile
2D Echocardiography	Normal chambers and valves. Ejection Fraction: 61%. No Regional wall motion abnormality, no valvular heart disease, no clot or vegetation
Ultrasound of whole abdomen and pelvis	No significant abnormality detected
Electromyography	Normal pattern

**Figure 1:**
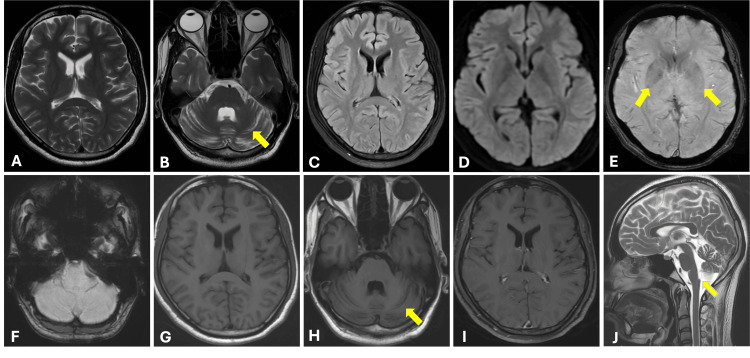
Axial T2-weighted images (A, B) and fluid-attenuated inversion recovery (FLAIR) image (C) show no abnormal signal intensity in the basal ganglia (A, C), with associated cerebellar atrophy (arrow in B). Axial diffusion-weighted imaging (DWI) image (D) demonstrates no restricted diffusion. Axial susceptibility-weighted imaging (SWI) images (E, F) show mild blooming in the bilateral lentiform nuclei (arrows in E) with no blooming foci in the cerebellum (F). Axial T1-weighted images (G, H) reveal normally appearing basal ganglia with mild cerebellar atrophy (arrow in H). Axial post-contrast T1-weighted image (I) shows no abnormal enhancement. Sagittal T2-weighted image (J) demonstrates clival hypertrophy (arrow).

Magnetic resonance imaging (MRI) of the brain revealed mild cerebellar atrophy and claval hypertrophy in the medulla. There was no abnormal T2/fluid-attenuated inversion recovery (FLAIR) signal intensity or post-contrast enhancement in the basal ganglia or cerebellum. Susceptibility-weighted imaging (SWI) showed mild blooming in bilateral lentiform nuclei and no blooming in the cerebellum (Figure 1).

Whole-exome sequencing revealed a heterozygous missense variant c.2222G>A in Exon 16 of the PLA2G6 gene that resulted in the amino acid substitution p.Arg741Gln. This variant has been classified as pathogenic according to the American College of Medical Genetics and Genomics (ACMG) guidelines. The patient was managed with levodopa (100 mg) and carbidopa (25 mg) thrice daily, trihexyphenidyl 2 mg thrice daily and baclofen 10 mg thrice daily. He remains under follow-up.

## Discussion

We present a 21-year-old male with rapidly progressive parkinsonism, spasticity, cognitive decline, and focal right-hand dystonia, harbouring a heterozygous pathogenic PLA2G6 variant (c.2222G>A; p.Arg741Gln). These findings align with PLA2G6-associated neurodegeneration (PLAN), likely representing atypical neuroaxonal dystrophy (aNAD) or PLA2G6-related dystonia-parkinsonism (PARK14) [3,11].

The patient’s rapid clinical deterioration, marked by bradykinesia, rigidity, postural instability, and loss of functional independence within eight months, reflects the more aggressive end of the PLAN spectrum. Magrinelli et al. (2022) described a similarly rapid deterioration in adult-onset PLAN with pyramidal signs and cognitive impairment [3]. Cognitive impairment, with prominent memory deficits and behavioural changes, also stands out. While Gregory et al. (2008) [2] reported neuropsychiatric features in adult-onset PLAN and Hanna Al-Shaikh et al. (2022) observed cognitive impairment in nearly half of PLAN patients, the combination of early behavioural changes and marked memory involvement is relatively uncommon [4]. The absence of generalised dystonia and ataxia deviates from typical PARK14 or aNAD presentations, further underscoring phenotypic variability [3,11].

Neuroimaging revealed cerebellar atrophy, claval hypertrophy -features more often associated with infantile forms of PLAN but less frequently described in adult-onset disease [3]. Mild basal ganglia blooming on SWI, without significant iron deposition, adds to this atypical profile. Magrinelli et al. reported that only one-third of PARK14 cases had MRI-detectable iron accumulation, supporting this atypical presentation [3].

Although heterozygous PLA2G6 variants have been described in a minority of PLAN cases, autosomal recessive inheritance remains the predominant pattern [12]. This may suggest an undetected second mutation or variable expressivity in heterozygous cases. In our patient, none of the siblings, parents, or grandparents were affected, suggesting either a de novo variant or incomplete penetrance. However, parental sequencing and extended analyses such as whole-gene or mRNA sequencing could not be performed due to financial constraints. Thus, the possibility of an undetected second pathogenic variant (e.g., intronic or regulatory) cannot be excluded, and this remains a limitation of our report. Ferese et al. (2018) reported a heterozygous PLA2G6 mutation with parkinsonism and rapid progression, similar to this case [12]. Paisán-Ruíz et al. (2009) also linked p.Arg741Gln to dystonia-parkinsonism with rapid progression [11]. PLA2G6 dysfunction, impairing membrane remodelling and mitochondrial regulation, likely drives neurodegeneration, even without prominent iron accumulation [2].

Levodopa provided partial benefit, consistent with variable responsiveness reported in PARK14 [3,11]. However, longer follow-up and quantitative assessment of treatment response are needed to better characterise the therapeutic response in this patient.

## Conclusions

This case highlights a rare constellation of rapidly progressive parkinsonism, spasticity, focal dystonia, cognitive decline, and atypical neuroimaging in association with a heterozygous p.Arg741Gln variant. Such presentations broaden the PLAN phenotype and the recognised clinical and radiological spectrum of PLAN and emphasise the value of early genetic testing in young adults with progressive spastic parkinsonism and unusual imaging findings.
